# Dengue score as a diagnostic predictor for pleural effusion and/or ascites: external validation and clinical application

**DOI:** 10.1186/s12879-018-2996-x

**Published:** 2018-02-23

**Authors:** Suhendro Suwarto, Mohammad Jauharsyah Hidayat, Bing Widjaya

**Affiliations:** 1 Tropical and Infectious Diseases Consultant, Pondok Indah Hospital, Jakarta, Indonesia; 2 Division of Tropical and Infectious Diseases, Department of Internal Medicine, Faculty of Medicine Universitas Indonesia, Cipto Mangunkusumo National Hospital, Jakarta, Indonesia; 3 Department of Radiology, Pondok Indah Hospital, Jakarta, Indonesia; 4 Department of Clinical Pathology, Pondok Indah Hospital, Jakarta, Indonesia

**Keywords:** Clinical application, Dengue score, External validation

## Abstract

**Background:**

The Dengue Score is a model for predicting pleural effusion and/or ascites and uses the hematocrit (Hct), albumin concentration, platelet count and aspartate aminotransferase (AST) ratio as independent variables. As this metric has not been validated, we conducted a study to validate the Dengue Score and assess its clinical application.

**Methods:**

A retrospective study was performed at a private hospital in Jakarta, Indonesia. Patients with dengue infection hospitalized from January 2011 through March 2016 were included. The Dengue Score was calculated using four parameters: Hct increase≥15.1%, serum albumin≤3.49 mg/dL, platelet count≤49,500/μL and AST ratio ≥ 2.51. Each parameter was scored as 1 if present and 0 if absent. To validate the Dengue Score, goodness-of-fit was used to assess calibration, and the area under the receiver operating characteristic curve (AROC) was used to assess discrimination. Associations between clinical parameters and Dengue Score groups were determined by bivariate analysis.

**Results:**

A total of 207 patients were included in this study. The calibration of the Dengue Score was acceptable (Hosmer-Lemeshow test, *p* = 0.11), and the score’s discriminative ability was good (AROC = 0.88 (95% CI: 0.83–0.92)). At a cutoff of ≥2, the Dengue Score had a positive predictive value (PPV) of 79.03% and a negative predictive value (NPV) of 90.36% for the diagnostic prediction of pleural effusion and/or ascites. Compared with the Dengue Score ≤ 1 group, the Dengue Score = 2 group was significantly associated with hemoconcentration> 20% (*p* = 0.029), severe thrombocytopenia (p = 0.029), and increased length of hospital stay (*p* = 0.003). Compared with the Dengue Score = 2 group, the Dengue Score ≥ 3 group was significantly associated with hemoconcentration> 20% (*p* = 0.001), severe thrombocytopenia (*p* = 0.024), severe dengue (*p* = 0.039), and increased length of hospital stay (*p* = 0.011).

**Conclusion:**

The Dengue Score performed well and can be used in daily practice to help clinicians identify patients who have plasma leakage associated with severe dengue.

## Background

Identifying plasma leakages in patients with dengue, which is also known as dengue hemorrhagic fever (DHF), is essential for determining which patients are at high risk for developing severe dengue [1]. However, over-diagnosis results in unnecessary hospital admission [2]. According to World Health Organization criteria, plasma leakage is defined as hematocrit (Hct) elevation of ≥20%, hypoalbuminemia, and the presence of pleural effusion/ascites [3]. Recently, Suwarto et al. [4] developed criteria for diagnosing pleural effusion and/or ascites as a marker of plasma leakage which involves four diagnostic variables: hemoconcentration≥15.1%, albumin concentration ≤ 3.49 mg/dL, platelet count ≤49,500/μL, and aspartate aminotransferase (AST) ratio ≥ 2.51. This diagnostic predictor is named the Dengue Score. This diagnostic prediction model has exhibited good calibration and discrimination [4]. The main differences between the Dengue Score and World Health Organization criteria are that the former has a lower hemoconcentration cutoff and includes two additional laboratory parameters (AST ratio and platelet count) as indicators of plasma leakage. Although this score may improve identifying plasma leakage in patients with dengue infection, it has not been validated. In fact, differences in patient characteristics could potentially influence this score’s diagnostic predictions. Therefore, external validation is needed to confirm the extent of the metric’s generalizability [5, 6].

One characteristic of an ideal scoring system is applicability in clinical practice [6]. In dengue-infected patients, plasma leakage is associated with hemoconcentration≥20%, severe thrombocytopenia, severe dengue, and increased length of stay (LOS) in the hospital. The first three variables significantly impact the management of dengue infection [7–9]. Therefore, we conducted a study to validate the Dengue Score and assess its clinical application in daily practice.

## Methods

A retrospective study was conducted in the ward of Pondok Indah Hospital in Jakarta, Indonesia. Patients≥14 years of age with acute fever and a positive non-structural protein 1 (NS-1) antigen test (SD BIOLINE Dengue Duo, Standard Diagnostics, Korea) who were admitted to the hospital from January 2011 through March 2016 were included in this investigation. Data were obtained from each patient’s medical records. The following clinical parameters were recorded: patient characteristics, hospital LOS, daily complete blood count, serum albumin, AST, and abdominal ultrasonography (USG) findings. The abdominal USG result was used to assess the presence or absence of pleural effusion and/or ascites. Serum albumin, AST, and abdominal USG examinations were performed 1–2 days after defervescence. Hct was calculated using published formulae [4]. The AST ratio was calculated by dividing the AST value by the upper bound of the reference range (which was 37 U/L in this investigation) [4]. Patients who were pregnant or had incomplete documentation were excluded.

The Dengue Score was calculated using four parameters: Hct increase≥15.1%, serum albumin≤3.49 mg/dL, platelet count≤49,500/μL and AST ratio ≥ 2.51. Each parameter was scored as 1 if present and 0 if absent [4]. Subjects were classified into three groups based on their Dengue Score: the Dengue Score ≤ 1 group, the Dengue Score = 2 group, and the Dengue Score≥3 group.

Severe thrombocytopenia was defined as a platelet count< 10,000/μL [8]. Severe dengue was defined as the presence of one of the following conditions: dengue shock syndrome (DSS), respiratory distress, severe bleeding, AST ≥ 1000 U/L, or organ failure [7]. LOS was calculated by determining the interval between a patient’s discharge and admission dates; values for LOS were classified into the categories of ≤5 days and > 5 days [9].

### Statistical analysis

The sample size was based on the required sample size to externally validate a prognostic model, namely, a minimum of 100 events (in this study, event refers to patients with pleural effusion and/or ascites) [10]. Thus, the minimum total sample size for this study was 200 patients. The performance of the Dengue Score was assessed for calibration and discrimination.

### Calibration

Calibration refers to the agreement between observed and expected outcomes (in this study, pleural effusion and/or ascites). Calibration was analyzed using the Hosmer-Lemeshow goodness-of-fit statistic, with *p* > 0.05 considered indicative of a well-calibrated score.

### Discrimination

The ability of the Dengue Score to discriminate between those with and without pleural effusion and/or ascites was assessed using the area under the receiver operating characteristic curve (AROC). AROC values> 0.8 were regarded as indicative of good discrimination.

### Clinical application of dengue score

The associations of clinical parameters (hemoconcentration≥20%, severe thrombocytopenia, severe dengue, and LOS) between the Dengue Score ≤ 1 and Dengue Score = 2 groups, between the Dengue Score ≤ 1 and Dengue Score≥3 groups, and between the Dengue Score = 2 and Dengue Score≥3 groups were analyzed using chi-square tests. All statistical analyses were performed using STATA, version 14.0 (StataCorp, College Station, TX, USA), and GraphPad Prism, version 7.00 for Windows (GraphPad Software, La Jolla, CA, USA).

## Results

A total of 207 patients with dengue infection satisfied our inclusion criteria. Clinical characteristics of these patients are presented in Table 1. Median fever onset of the patients on the admission day was 3 (Interquartile range [IQR], 2–4). Table 2 shows a comparison of fever onset and the clinical parameters of the Dengue Score for each study group.

**Table 1:** Patient characteristics and clinical parameters

Characteristic	Number of patients (N = 207)
Age^a^	33 (23–46)
Sex, n (%)	
Male	91 (44)
Female	116 (56)
Dengue score, n (%)	
0	37 (17.9)
1	46 (22.2)
2	38 (18.4)
3	55 (26.6)
4	31 (15.0)
Hemoconcentration, n (%)	
≤ 20%	172 (83.1)
> 20%	35 (16.9)
Severe thrombocytopenia, n (%)	
≥10,000/μL	182 (87.9)
< 10,000/μL	25 (12.1)
Severe dengue, n (%)	
AST ≥ 1000	5 (2.4)
DSS	4 (1.9)
Respiratory distress	4 (1.9)
Severe bleeding	3 (1.4)
Organ failure	3 (1.4)
Length of stay, n (%)	
≤ 5 days	101 (48.8)
> 5 days	106 (51.2)
Abdominal USG, n (%)	
Absence of pleural effusion and/or ascites	101 (48.8)
Presence of pleural effusion and/or ascites	106 (51.2)

^a^Data presented as median (interquartile range)

**Table 2:** Comparison of patients with Dengue Score ≤ 1, Dengue Score = 2 and Dengue Score ≥ 3

	Dengue Score ≤ 1 (*N* = 83)	Dengue Score = 2 (*N* = 38)	Dengue Score ≥ 3 (*N* = 86)
Fever onset, days^a^	3 (2–4)	2.5 (2–4)	3 (2–3)
Hemoconcentration ≥15.1%, n (%)			
Negative	83 (100%)	31 (81.6)	36 (41.9)
Positive	0 (0%)	7 (18.4)	50 (58.1)
Serum albumin ≤3.49 mg/dL, n (%)			
Negative	68 (81.9)	21 (55.3)	8(9.3)
Positive	15 (18.1)	17 (44.7)	78 (90.7)
Platelet count ≤49,500, n (%)			
Negative	69 (83.1)	10 (26.3)	2 (2.3)
Positive	14 (16.9)	28 (73.7)	84 (97.7)
AST ratio ≥ 2.51, n (%)			
Negative	66 (79.5)	14 (36.8)	9 (10.5)
Positive	17 (20.5)	24 (63.2)	77 (89.5)

^a^Data are presented as the median (interquartile range)

### Performance of the dengue score

**Fig. 1:**
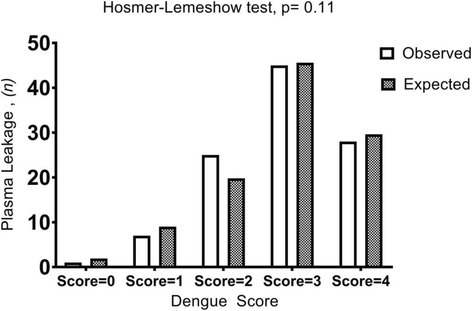
Calibration of the Dengue Score: Observed and expected pleural effusion and/or ascites (*N* = 207)

**Fig. 2:**
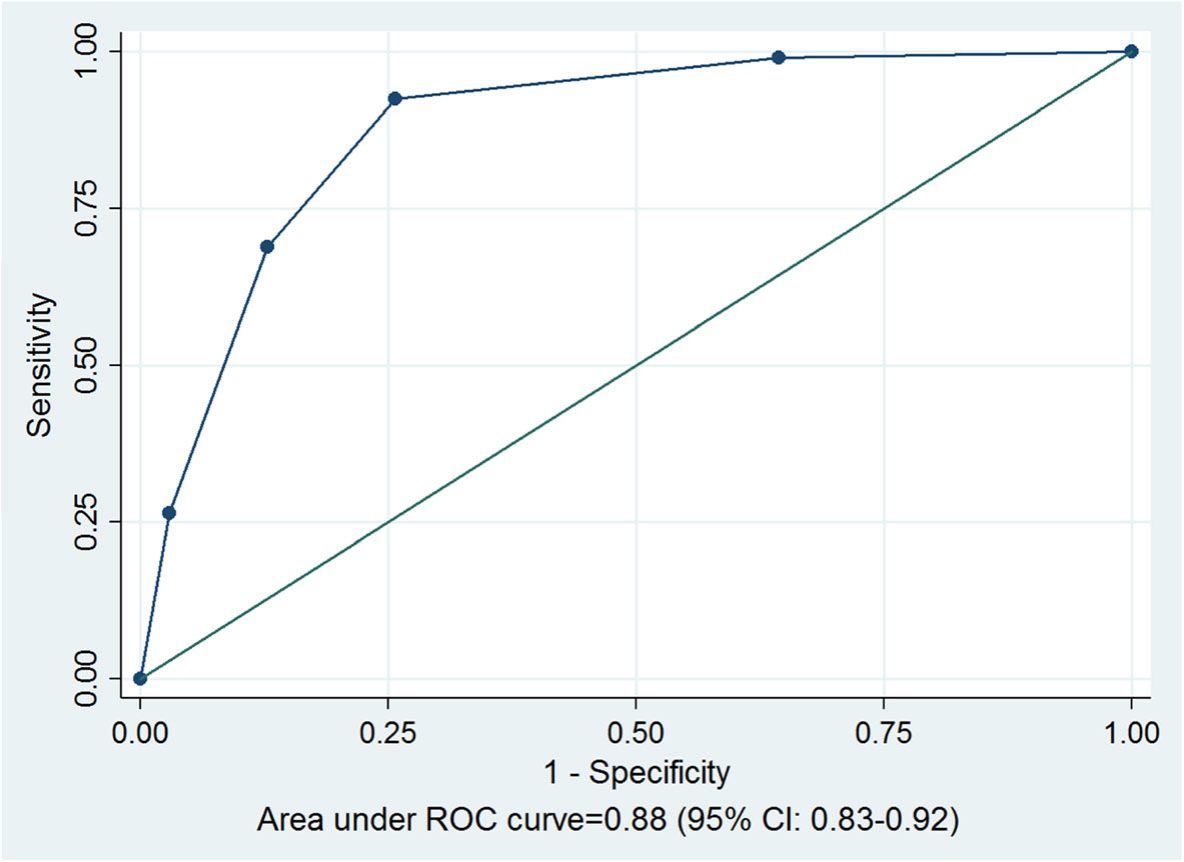
Area under the receiver operating characteristic (AROC) of the Dengue Score

The Hosmer-Lemeshow test indicated that the Dengue Score exhibited good calibration (*p* = 0.11) (Fig. 1), and the AROC indicated that this metric had good accuracy (0.88; 95% CI: 0.83–0.92)] (Fig. 2). With a cutoff of ≥2, the Dengue Score had a sensitivity of 92.45%, a specificity of 74.26%, a positive predictive value (PPV) of 79.03%, and a negative predictive value (NPV) of 90.36%; 83.57% of cases were correctly classified (Table 3).

**Table 3:** Sensitivity and specificity of different Dengue Score cutoff points

Cutoff score	Sensitivity	Specificity	Positive predictive value	Negative predictive value	Correctly classified
≥1	99.06%	35.64%	61.76%	97.30%	68.12%
≥2	92.45%	74.26%	79.03%	90.36%	83.57%
≥3	68.87%	87.13%	84.88%	72.73%	77.78%
≥4	26.42%	97.03%	90.32%	55.68%	60.87%

### Clinical application

Relative to the Dengue Score ≤ 1 group, the Dengue Score = 2 group was significantly associated with hemoconcentration≥20% (*p* = 0.029), severe thrombocytopenia (p = 0.029), and increased LOS (*p* = 0.003). Relative to the Dengue Score ≤ 1 group, the Dengue Score ≥ 3 group was significantly associated with hemoconcentration> 20%, severe thrombocytopenia, severe dengue, and increased LOS (*p* < 0.001 for each comparison). Finally, compared with the Dengue Score = 2 group, the Dengue Score ≥ 3 group was significantly associated with hemoconcentration> 20% (*p* = 0.001), severe thrombocytopenia (*p* = 0.024), severe dengue (*p* = 0.039), and increased LOS (*p* = 0.011) (Table 4).

**Table 4:** Associations between clinical parameters and Dengue Score groups

	Dengue Score ≤ 1	Dengue Score = 2	Dengue Score ≥ 3
Hemoconcentration, n (%)			
≤ 20%	83 (100)	35 (92.1)^a^	54 (62.8)^a, b^
> 20%	0 (0)	3 (7.9)	32 (37.2)
Severe thrombocytopenia, n (%)			
≥10,000/μL	83 (100)	35 (92.1)^a^	64 (74.4)^a, b^
< 10,000/μL	0 (0)	3 (7.9)	22 (25.6)
Severe dengue, n (%)			
Negative	83 (100)	36 (94.7)	69 (80.2)^a, b^
Positive	0 (0)	2 (5.3)	17 (19.8)
Length of stay, n (%)			
≤ 5 days	62 (74.7)	18 (47.4)^a^	21 (24.4)^a, b^
> 5 days	21 (25.3)	20 (52.6)	65 (75.6)

Data are presented as number of patients (%) for each group. Chi-square tests were used to assess associations between findings for two groups. ^a^ a significant association relative to the Dengue Score ≤ 1 group; ^b^ a significant association relative to the Dengue Score = 2 group (*p* < 0.05)

## Discussion

In this study, the Dengue Score was validated using an external data set. The laboratory parameters, i.e., levels of AST, serum albumin and USG, were assessed in the critical phase. The degree of hemoconcentration and the lowest platelet count were determined based on daily complete blood count measurement. A previous study reported that an elevated AST level, a lower albumin concentration, the hematocrit peak, the lowest platelet count, and increased detectable of pleural effusion/ascites by USG were found in the critical phase or 1–2 days after defervescence [4, 11, 12]. In addition, the degree of hemoconcentration can be calculated by using the minimum hematocrit during admission or the hematocrit at convalescence as a reference [12]. In addition, a cut-off point for platelet count< 50,000/μL and serum albumin< 3.5 g/dL are widely used as indicators of plasma leakage [13, 14]. Furthermore, using the degree of hemoconcentration and AST ratio will resolve the problem of differences in reference limits among laboratories. Therefore, these laboratory parameter cut-off points can be used universally in various laboratories [4, 12].

Performance analysis of the Dengue Score using the Hosmer-Lemeshow test produced nonsignificant results, indicating that the score is well calibrated; this metric has good discriminative ability, as revealed by an AROC> 0.8. When a cutoff of ≥2 was used, the Dengue Score exhibited higher sensitivity (92.45% vs 82.47%) and similar specificity (74.26% vs 70.42%) to that reported in a prior study [4]. In addition, compared with our previous investigation, this study found a higher NPV (90.36% vs 74.63%) and a similar PPV (79.03% vs 79.21%) for the Dengue Score [4]. A score with high sensitivity is useful for screening to identify a disease, and a negative result can be useful to exclude a disease (in this case, plasma leakage or DHF) [15, 16]. Our findings are consistent with clinical parameters demonstrating that patients with a Dengue Score ≤ 1 did not develop hemoconcentration> 20%, severe thrombocytopenia, or severe dengue. Therefore, patients with Dengue Score ≤ 1 are likely to have dengue fever (DF), the mildest form of dengue infection.

Clinical parameter analyses demonstrated that compared with the Dengue Score ≤ 1 group, the Dengue Score = 2 group was significantly associated with hemoconcentration≥20%, severe thrombocytopenia, and increased LOS. Hematocrit increases of 20% or more are regarded as indicative of increased vascular permeability, which can result in plasma leakage and hypovolemia [7]. Dengue-infected patients with a rise in hemoconcentration≥20% should immediately receive intravenous fluids [3, 7]. Prior studies have indicated that degree of thrombocytopenia correlated with severity of plasma leakage and length of hospital stay [3, 9, 17]. Another study reported that the proportion of patients with severe thrombocytopenia was significantly higher in DHF group compare to DF group (42.9% vs 0%) [18]. Previous findings and our clinical parameter analysis results support the diagnostic prediction that patients with a Dengue Score = 2 have a high probability of plasma leakage and may therefore should be closely observed in health care facilities.

We also found that relative to the Dengue Score ≤ 1 and =2 group, the Dengue Score ≥ 3 group was significantly associated with hemoconcentration> 20%, severe thrombocytopenia, severe dengue, and increased LOS. A previous study revealed that 90% of DSS group subjects had hemoconcentration> 20% compared to 67% in DHF group subjects [19]. This finding was in accordance with another study reporting a significantly higher proportion of hemoconcentration> 20% in a severe dengue group compared to a non-severe dengue group [12]. Fernando et al. [20] reported that the proportion of severe thrombocytopenia patients was significantly higher in severe dengue group compare to non-severe dengue group (68.18% vs 3.03%). In addition, severe thrombocytopenia was significantly associated with the development of severe dengue [21]. These findings may explain the higher proportion of hemoconcentration> 20% and severe thrombocytopenia in the Dengue Score ≥ 3 group relative to the Dengue Score ≤ 1 and =2 group due to a significantly higher proportion of severe dengue in the former.

Hypoalbuminemia, one parameter of the Dengue Score, has been reported to be a cause of fluid accumulation, and this condition could be used as a surrogate marker for severe plasma leakage [22, 23]. Thein et al. [24] reported that a combination of three variables, i.e., hemoconcentration, rapid decrease in platelet count, and fluid accumulation, had high specificity for predicting severe dengue. Trung et al. [25] demonstrated elevated AST levels to be correlated with disease severity, as indicated by manifestations such as vascular leakage and bleeding. Another study reported that elevated AST concurrent with hypoalbuminemia and hemoconcentration concurrent with thrombocytopenia could be used to identify severe dengue [26]. In addition, a study of dengue patients hospitalized between 2008 and 2013 showed that a combination of three variables, namely, fluid accumulation, elevated AST level and thrombocytopenia, were associated with severe dengue [27], and a meta-analysis study reported that four laboratory parameter variables, hemoconcentration, hypoalbuminemia, elevated AST level and thrombocytopenia, were significantly associated with DSS [28]. These prior findings may explain why the group of subjects with a minimum combination of three factors among hemoconcentration, hypoalbuminemia, thrombocytopenia and elevated AST ratio, that is, the Dengue Score ≥ 3 group, was more closely associated with severe dengue and prolonged hospital stay than the Dengue Score ≤ 1 and =2 group.

This study has a limitation. It used a retrospective design, and there was therefore the potential for bias in data collection. We only included patients with complete data, which we defined as including abdominal USG findings. Therefore, the study subjects may have represented a relatively ill patient population.

## Conclusions

As a diagnostic predictor of pleural effusion and/or ascites, the Dengue Score is well calibrated and exhibits good discrimination. The application of this simple scoring system, which comprises laboratory parameters that are routinely measured in clinical practice, may help clinicians identify patients with plasma leakage associated with severe dengue.

## Abbreviations

AROC: area under the receiver operating characteristic curve
AST: aspartate aminotransferase
DF: dengue fever
DHF: dengue hemorrhagic fever
DSS: dengue shock syndrome
Hct: hematocrit
LOS: length of stay
NPV: negative predictive value
NS-1: non-structural protein 1
PPV: positive predictive value
USG: ultrasonography
